# Practical metrics for establishing the health benefits of research to support research prioritisation

**DOI:** 10.1136/bmjgh-2019-002152

**Published:** 2020-08-31

**Authors:** Beth Woods, Laetitia Schmitt, Claire Rothery, Andrew Phillips, Timothy B Hallett, Paul Revill, Karl Claxton

**Affiliations:** 1 Centre for Health Economics, University of York, York, Yorkshire, UK; 2 Institute for Global Health, University College London, London, UK; 3 Department of Infectious Disease Epidemiology, Imperial College London, London, London, UK

**Keywords:** HIV, health economics, health services research

## Abstract

**Introduction:**

We present practical metrics for estimating the expected health benefits of specific research proposals. These can be used by research funders, researchers and healthcare decision-makers within low-income and middle-income countries to support evidence-based research prioritisation.

**Methods:**

The methods require three key assessments: (1) the current level of uncertainty around the endpoints the proposed study will measure; (2) how uncertainty impacts on the health benefits and costs of healthcare programmes and (3) the health opportunity costs imposed by programme costs. Research is valuable because it can improve health by informing the choice of which programmes should be implemented. We provide a Microsoft Excel tool to allow readers to generate estimates of the health benefits of research studies based on these three assessments. The tool can be populated using existing studies, existing cost-effectiveness models and expert opinion. Where such evidence is not available, the tool can quantify the value of research under different assumptions. Estimates of the health benefits of research can be considered alongside research costs, and the consequences of delaying implementation until research reports, to determine whether research is worthwhile. We illustrate the method using a case study of research on HIV self-testing programmes in Malawi. This analysis combines data from the literature with outputs from the HIV synthesis model.

**Results:**

For this case study, we found a costing study that could be completed and inform decision making within 1 year offered the highest health benefits (67 000 disability-adjusted life years (DALYs) averted). Research on outcomes improved population health to a lesser extent (12 000 DALYs averted) and only if carried out alongside programme implementation.

**Conclusion:**

Our work provides a method for estimating the health benefits of research in a practical and timely fashion. This can be used to support accountable use of research funds.

**Key questions**

**What is already known?**

- Methods are available to estimate the value of research studies but are not widely understood, appreciated or applied.

**What are the new findings?**

- We provide a method and companion Microsoft Excel tool that can be used to estimate the health benefits of research studies without using advanced value of information methods.
- The tool can be populated using a range of evidence or used to test how different assumptions affect the value of research.
- We illustrate the method by applying it to estimate the value of research studies on HIV self-testing programmes.

**What do the new findings imply?**

- Our work provides a method for estimating the health benefits of research in a practical and timely fashion; these estimates can be considered alongside research costs to prioritise research studies for funding.

## Introduction

Globally, significant resource and effort is spent on health-related research with the 10 largest public and philanthropic funders spending US$37.1 billion in 2013.^1^ An important component of this funding is dedicated to basic science and preclinical research. However, much research aims to better understand current epidemiological patterns, healthcare provision and patient outcomes, and how they would be impacted by alternative interventions with a view to informing healthcare investments in the near-term. Clinical trials, surveillance programmes, cost studies, morbidity surveys and implementation studies all serve this purpose. By improving the information available to support investment decisions, they have the potential to improve population health. However, research is costly and those funding research have constraints on their ability to expand research budgets. This raises the question of which research activities should be prioritised.

To answer this question there is a need to understand why evidence is valuable to healthcare systems and the populations they serve, and how to assess the value of specific research proposals. This has been recognised by a number of stakeholders and a set of methods called value of information analysis allow the value of specific research proposals to be quantified.^2–4^ Value of information analysis has been applied in a range of contexts in high-income settings, for example, to assess the value of clinical trials of interventions for which limited evidence exists.^5 6^ Previous studies have also estimated the value of further research in low-income and middle-income countries (LMICs).^4 7 8^ These studies used advanced methods^4 7 8^ that require specific types of analyses to have been conducted (probabilistic analyses of a model already addressing the policy question of interest).^9 10^ The application of value of information analysis to help prioritise research has, therefore, been limited as the advanced methods required are often not practical given time and resource constraints, and computation may be impractical where transmission models are required to represent disease dynamics.

In this paper, we use a graphical method and simple metrics to show how the principles of value of information analysis can be applied in these common but challenging circumstances. We provide a simple excel tool to facilitate use of the method and explain how the method can be applied using different types of evidence including typical outputs from existing cost-effectiveness models. We also discuss how this type of analysis can inform key policy questions relating to the allocation of research funds. We then apply this method in a case study assessing the value of research in HIV self-testing programmes in Malawi.

The methods presented are relevant to any party with a stake in ensuring health research funds are used in a way that is expected to improve population health. This includes research funders, researchers and healthcare decision-makers within LMICs who rely on robust evidence to make investment decisions. The latter group includes individuals within ministries of health charged with prioritising health programmes (including designing health benefits packages), and other decision-makers at a regional and national level who are responsible for healthcare resource allocation. The methods presented apply where a single budget is used to fund research and service provision, and to the more common situation where budgets for these activities are separate.

## Methods

### Graphical illustration using a simple quantitative tool to quantify the value of research

Cost-effectiveness analyses are routinely used to assess whether a programme is expected to improve population health once the health opportunity costs imposed by additional programme spending are accounted for. This assessment can be summarised using an estimate of the net disability-adjusted life years (DALYs) averted by the programme. This reflects both the health benefits of the programme and an assessment of the health forgone as funding a programme means that resources will be unavailable for the delivery of other programmes. This is calculated as the DALYs directly averted via the programme minus the DALYs incurred elsewhere in the health system due to the additional programme funding required.

In the same way, we can quantify the net DALY impact of investing in healthcare provision, we can also quantify the net DALY impact of investing in research. This idea is the basis for value of information analysis.

**Figure 1:**
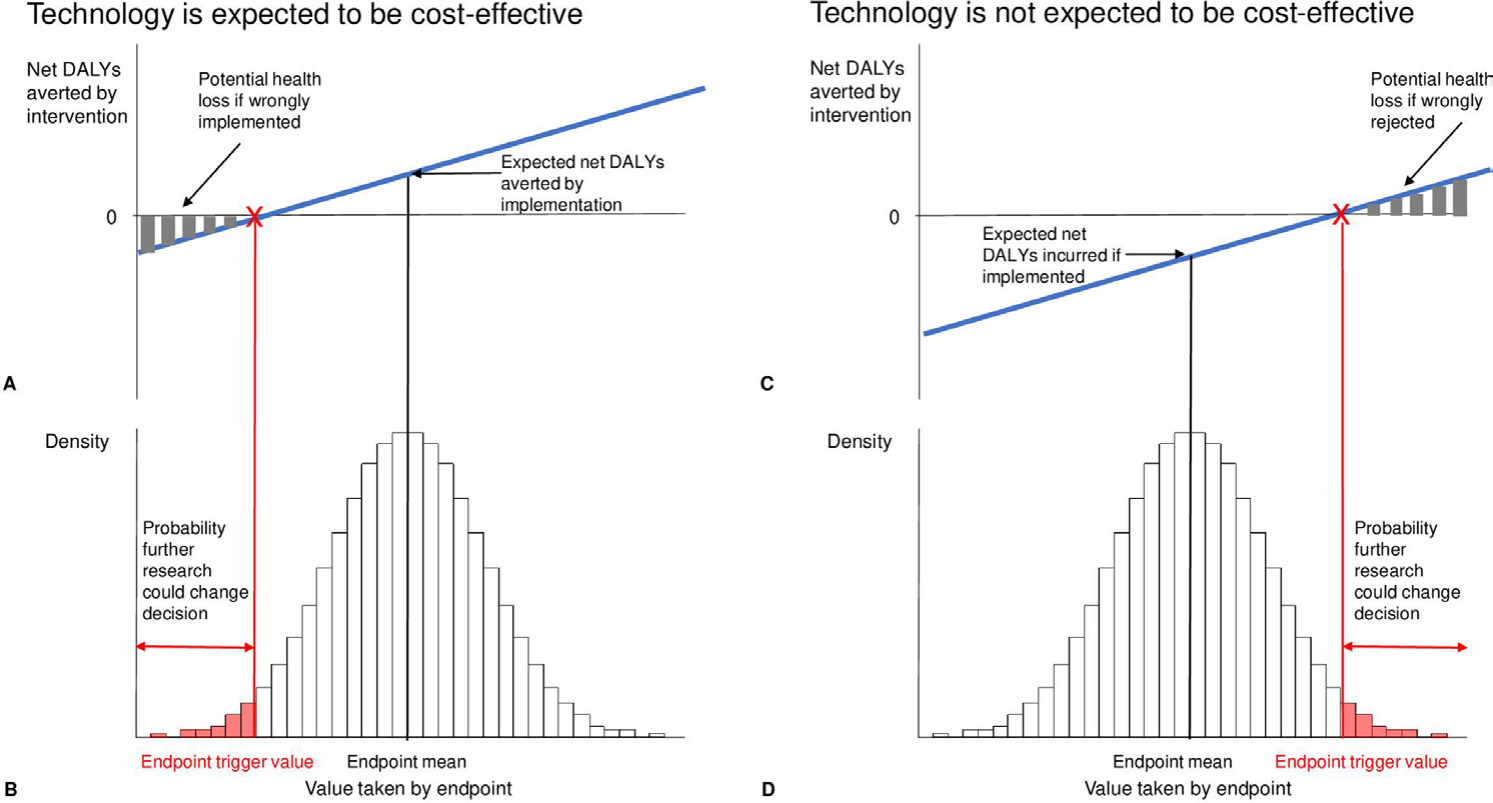
Calculating the net health effects of research. Legend: (A) shows net disability-adjusted life years (DALYs) averted by the programme for different values of the endpoint of interest when the programme is expected to be cost-effective based on current evidence; (B and D) show the prior on the uncertain endpoint; (C) shows net DALYs averted by the programme for different values of the endpoint of interest when the programme is not expected to be cost-effective based on current evidence.

To assess the value of a research study or other data collection or evidence gathering activities, we need to understand the types of uncertainty that we could examine in a study with particular endpoints. These endpoints may be epidemiological, clinical, patient reported, process related or economic. For example, we might be uncertain about the effectiveness of a drug, the uptake of a rural community-based prevention programme, the quality of life of people with different treatment outcomes, or the cost of implementing a new diagnostic pathway. To assess the value of improving information relating to an endpoint, we need to understand our current level of uncertainty about the endpoint given existing evidence. This uncertainty can be described by a probability distribution showing the likelihood that the endpoint takes different values. This distribution is often called a prior, since it is based on existing knowledge of uncertainty about the specific endpoint. Figure 1B shows the prior on an uncertain endpoint as a histogram.

Uncertainty about the endpoint alone is not sufficient to justify expenditure on research. For research to deliver value, the uncertainty in the endpoint must translate to uncertainty about whether the programme is cost-effective. For example, we might be highly uncertain about a programme’s effects on clinical outcomes. However, if the programme is cost-effective across the range of plausible clinical outcomes then further research on this endpoint may not deliver value in this setting as it would not change the decision about funding the intervention.

We can assess whether uncertainty in the endpoint is likely to translate to uncertainty about cost-effectiveness by estimating the net DALYs we would expect to avert if the endpoint was found to take the different values reflected in the prior. This is shown in figure 1A. In this illustration, as the endpoint increases, the net DALYs averted increase. This reflects estimates of how both DALYs averted and additional costs (or cost savings) change with the value of the endpoint. It also reflects a measure of the health opportunity cost of financing the programme, as this allows the additional costs of the programme to be converted to health foregone.

The mean value of the endpoint represents our ‘best guess’ of the value the endpoint takes given currently available information. At this value the net DALYs averted by the programme are positive and the programme would be considered cost-effective. However, below a certain ‘trigger’ value of the endpoint, the net health effects of the programme become negative, that is, the programme is not cost-effective. The shaded area of the prior histogram (figure 1B) indicates the probability that the endpoint will fall below the trigger point. This is the probability that the intervention will turn out not to be cost-effective and that implementation will reduce population health. However, if we conduct research to improve our understanding of the endpoint this is the probability that the research could change the implementation decision. If it is considered implausible that the endpoint could take a value as extreme as the trigger point then further research will not result in a change in decision and, therefore, based on the available evidence, may not be considered an appropriate use of resources. This emphasises that we should care about uncertainty in endpoints when it leads to uncertainty in decisions.

Without additional research, on average implementation averts DALYs but if low values of the endpoint are realised, implementation reduces population health. If research is conducted and indicates that the endpoint falls below the trigger point (i.e. the programme is not cost-effective), then the programme will not be implemented. Research therefore avoids the health losses associated with programme implementation under these conditions as shown by the grey bars in figure 1A. These bars, therefore, represent the potential health gains from research. The expected net DALYs averted via research are calculated as the health gains (resulting from avoided health losses) when the endpoint takes values below the trigger point, that is, the shaded bars in figure 1A weighted by the probability of the quantity taking each value below the trigger point, that is, the shaded bars in figure 1B.

Figure 1C, D show how the value of research can be calculated when the programme is not expected to be cost-effective based on current information. Without further research the programme is not implemented and no population health gains are generated. With further research, there is a possibility that the endpoint will take values sufficiently high to support implementation and net DALYs are averted. The possible health gains from research are again shown by the grey bars (figure 1C).

This method shows the value of completely eliminating the uncertainty around the endpoint. Although in reality further research will not resolve all uncertainty, the estimates generated provide an expected upper bound for the population health benefits from research for the setting of interest.

We express the value of the research proposals using two different metrics. The first is the net DALYs averted by using the research to improve decision making. Where a research study is expected to be used in a number of countries, the approach described above can be applied for each country and the net DALYs averted across countries can be calculated. Individual country estimates of the net DALYs averted by research are likely to differ for a range of reasons including differences in the size of the population that stand to benefit from research, the costs and health benefits of the programme and the health opportunity costs of healthcare funds.

The net DALYs averted by research provides an estimate of the expected maximum population health gains from research accounting for both health gains and programme costs, but it does not consider research costs. Funding a specific research proposal has opportunity costs, which are the health gains that could be generated by using this funding for other research studies.

The second metric is, therefore, the maximum amount a research funder should be willing to spend on the research, given its estimated net health effects. This metric is estimated by multiplying the net DALYs averted by research by a measure of the opportunity cost of research funds. We assume that research funds have similar levels of opportunity costs as funds for service provision. For example, if a research study is expected to avert 1000 DALYs and our measure of opportunity costs indicates that every US$500 of expenditure results in an additional 1 DALY being incurred elsewhere in the health system, then the maximum a research funder should be willing to spend on the research would be US$500 000. If they spend more than this the health opportunity costs of funding the research would exceed 1000 DALYs and thus, more than outweigh the net health gains from research. Given the very different sources of funding that typically underpin service provision and research, the opportunity cost of research funds may differ from the opportunity cost of service funding. We will return to the question of how the opportunity cost of research funds could be estimated in the discussion.

**Figure 2:**
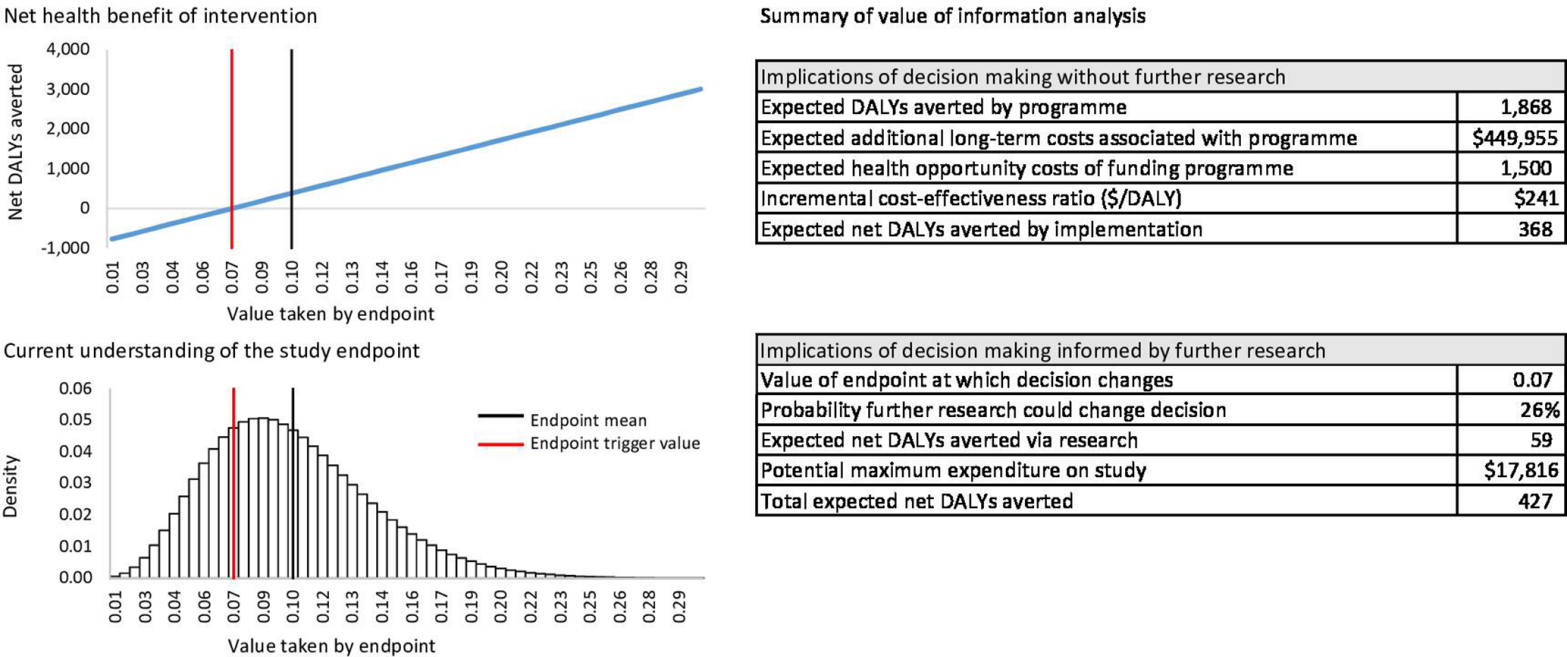
Output of quantitative excel tool for calculating the net health effects of research. DALYs, disability-adjusted life years.

To illustrate the approach, we use a numeric example where we are interested in an outcomes endpoint that can, in principle, take different values between 0 and 1 (eg, the probability of treatment response). Our existing knowledge of the endpoint indicates it is expected to take a value of 0.10 (SE 0.04, 95% CI 0.04, 0.19), which allows us to define its prior (we apply a beta distribution here). In a second step, we make use of existing information about how different values of the endpoint influence health effects and costs of the programme. In the present example, we know that if the endpoint takes the average value, the programme is expected to avert 2000 DALYs. If the endpoint takes the value at the lower bound of the CI the programme is expected to avert 1000 DALYs, whereas if the endpoint takes the value at the higher bound of the CI, the programme is expected to avert 3000 DALYs. The expected additional long-term cost associated with the programme is US$450 000 and is not expected to vary with the endpoint. Lastly, we evaluate the health opportunity cost associated with funding the intervention. This is 1500 DALYs based on additional costs of US$450 000 and an estimate of health opportunity cost of US$300/DALY. This information about the DALYs averted at different values of the endpoint, and about opportunity costs, allows us to estimate the net DALYs averted at different values of the endpoint. We provide a simple Microsoft Excel tool to allow users to review the numeric example and apply the approach to their own contexts. This tool is available in the online supplementary material, for the most up to date version of the tool see https://www.york.ac.uk/che/research/global-health/methods-guidelines/%23tab-4. The tool provides a graphical summary of the prior information and the relationship between net health effects and the endpoint of interest as shown in figure 2.

10.1136/bmjgh-2019-002152.supp1 Supplementary data

The tool uses regression methods to generate estimates of the net health effects of a programme at all plausible values of the endpoint. The regression uses estimates of DALYs averted and additional costs at different values of the endpoint that are entered by the user. Two regressions are then fitted, one regressing DALYs averted on the endpoint and the other regressing additional costs on the endpoint. Options are available to use linear regression, or to assume range of non-linear relationships between the endpoint and DALYs averted or additional costs.

The tool uses the data entered to generate estimates of the benefits of research. The tool shows the implications of making decisions based on current evidence, and the potential benefits of making decisions on the basis of further research as shown in figure 2. Without further research we can only base our decision on what we expect to occur. We expect that the programme averts 1868 DALYs (the expected health benefits (1868) are not identical to the health benefits at the mean value of the endpoint (2000) as the beta distribution used to describe the endpoint is not symmetrical) with a health opportunity cost of 1500 DALYs, that is, 368 net DALYs averted. On this basis, we implement the programme based on current evidence. If we conduct research, we will gain more information about which value the endpoint takes. If the endpoint is as expected or higher, there is no change to the decision. If the endpoint is lower than the trigger point of 0.07, the net DALYs averted become negative and we choose not to implement the intervention. Weighting the probability of observing values of the endpoint below 0.07 by the net DALYs averted by avoiding implementation, we expect the research to avert 59 DALYs. If the research is only considered relevant in this context then the maximum a research funder should be willing to spend on the research is US$17 800, suggesting that this may not be a high priority area for research. If the research is expected to inform decision making in other countries, then the process can be repeated for each country, and the value of research across countries can be calculated.

### Guidance for gathering evidence to inform estimates of the value of research

As shown above, a necessary part of any assessment of the value of research is formulating a view on the current level of uncertainty about the endpoints the research will examine. This uncertainty can be represented as a prior distribution. Evidence from existing studies including pilot studies or systematic reviews can be used to formulate priors. In practice, however, many research studies examine combinations of interventions and contexts which have not previously been studied. When evaluating a specific research proposal formally elicited expert opinion^11 12^ may, therefore, be valuable to complement quantitative and qualitative information to formulate priors.

It is also necessary to estimate how the health benefits and additional costs of the programme change with the endpoint. Where a cost-effectiveness model is available, this can be obtained by conducting one-way sensitivity analysis, that is, varying the values taken by the endpoint of interest and recording the corresponding variations in health benefits and additional long-term costs associated with the intervention. If a cost-effectiveness model is not available for the context of interest, or existing models cannot be easily adapted, then formal expert elicitation can be used to quantify the magnitude of health benefits and additional costs at different levels of the endpoint.

In order to estimate the net health effects of programmes, we require an understanding of how additional programme costs translate to health opportunity costs. Recent work has estimated the opportunity cost of domestic healthcare spending in a wide range of LMICs.^13^ Where programmes are funded via overseas aid the opportunity costs of this funding will depend on the remit of the funder. An understanding of the potential health opportunity cost of an overseas aid funding stream can be garnered by reviewing the cost-effectiveness of those interventions that are and are not currently funded, and potentially developing a cost-effectiveness league table of funded programmes.

Specification of each element described above is likely to require judgements regarding which evidence is relevant and how to use that evidence. By using the tool provided, users can explore the sensitivity of their results to each of these elements. In some contexts, the time-sensitive nature of a research-funding decision, analyst capacity or funding availability, may make it infeasible to assemble these types of evidence. In these contexts, the tool can provide a quantitative basis for testing how different assumptions influence both the net DALYs averted by the research and the maximum amount a funder should be willing to spend on the research.

We now show how the approach can be applied to a specific example. In this example, evidence is available from a cost-effectiveness model but no probabilistic sensitivity analysis has been conducted thus prohibiting use of standard value of information methods.

### Self-testing example using the HIV synthesis model

We show how these methods can be applied to assess the value of research in HIV self-testing programmes in Malawi. Self-testing programmes have been the subject of a number of recently published and ongoing research studies in sub-Saharan Africa (for some examples see refs. 14–18). We use the HIV synthesis model^19 20^ which has been used to assess the cost-effectiveness of a range of HIV prevention and treatment investments in different settings. The self-testing programme under evaluation is not currently part of the HIV investment strategy. We assess two possible scenarios to estimate the population health benefits from research studies on self-testing programmes. Under the first scenario, no research is conducted and investment in self-testing is based on current evidence about the costs and benefits of the programme. Under the second scenario, research is commissioned and the results of the research inform the decision about investment in self-testing.

Studies of HIV testing have included a range of endpoints measuring intervention effectiveness and costs at different points in the cascade of care. Frequently reported endpoints include coverage and uptake, HIV positivity, linkage and retention in care, and programme costs.^18^ The cost-effectiveness of self-testing is strongly linked to the cost per new HIV diagnosis^21^ which is calculated as the programme cost per person divided by the proportion of people diagnosed with HIV as a result of the programme. This suggests that two endpoints: programme costs and the proportion of people diagnosed with HIV, are likely to be important determinants of whether testing is cost-effective and therefore important targets for further research. The proportion of people diagnosed with HIV within facility-based care as a proportion of those targeted for testing reflects the combined effect of multiple endpoints collected within testing studies such as uptake, HIV positivity within those tested and linkage to facility-based care. We, therefore, examine a cost study focused on the cost of the self-testing programme per individual eligible for testing; and an outcomes study estimating the proportion of the eligible population who are diagnosed with HIV in facility-based care.

To evaluate the research proposals, we require priors describing the uncertainty about both programme costs and the proportion of the eligible population who are diagnosed with HIV in facility-based care. These priors will depend on the characteristics of the target population and implementation setting, the details of the testing programme such as whether measures to enhance linkage are proposed (eg, financial incentives, community-based support) and other contextual factors. The priors will, therefore, depend on the exact details of a specific research proposal and are most likely best formulated by combining available data, qualitative information and expert opinion. For the purposes of this demonstration, we use only data from the literature to inform the priors. We use data from a systematic review and meta-analysis,^18^ focusing on those data relating to self-testing. This work reflects the fairly limited data on self-testing available in 2015, when many of the self-testing studies were designed. For further details see online supplementary material S1.

Estimates of the additional costs and DALYs averted by a self-testing programme were derived from the HIV synthesis model. This is an individual-based stochastic model of heterosexual transmission, progression and treatment of HIV infection. We used outputs from the model generated by the ‘Working group on cost effectiveness of HIV testing in low income settings in sub-Saharan Africa’^21^ which examined the effects of expanding HIV testing beyond a core testing programme considered to represent current standard of care in many countries. This core testing programme included testing for: pregnant women, symptomatic individuals, female sex workers (although this is not fully implemented in many countries) and men coming forward for circumcision. This work examined the relationship between cost per HIV diagnosis and long-term cost effectiveness. The demographics of the population and the HIV epidemic features were based on those for Malawi and the model is calibrated to data that are representative of this setting. This work examined the cost-effectiveness of testing for a wide range of scenarios. The scenarios reflect variation in the expanded testing programme testing rates, how well the programme targets HIV positive individuals and cost per test. The scenarios also reflect uncertainty about the context in which the programme is implemented in terms of the nature of the epidemic, ART programme characteristics and the core testing programme. The model time horizon was 50 years and a discount rate of 3% was used for costs and outcomes.

We used the scenario analysis outputs from the model to estimate the relationship between costs and DALYs averted and both endpoints of interest (the proportion of the targeted population diagnosed with HIV in facility-based care and programme costs). For further details, see online supplementary material S2.

Estimating the net DALYs averted by self-testing, requires a measure of the health opportunity cost of the funds used to pay for self-testing. We have used a measure of opportunity cost of US$500/DALY. This represents the cost per DALY averted of those services we expect to be displaced by investments in self-testing. US$500/DALY is considered a relevant cost-effectiveness threshold for resource allocation within the HIV programme which is overwhelmingly reliant on overseas aid.^21 22^ Additionally, HIV investments which Malawi and other countries in sub-Saharan Africa have struggled to scale up often have incremental cost-effectiveness ratio (ICERs) around US$500/DALY, and HIV budgets have been shown to be exhausted in South Africa after funding interventions with ICERs around US$500/DALY.^23^ Where delivery of HIV interventions draws on resources that would otherwise be used for non-HIV health activities a lower threshold is more appropriate, we return to this in the discussion.

The analysis of the outputs from the HIV synthesis model were conducted in the statistical software R and associated packages.^24–39^

## Results

**Figure 3:**
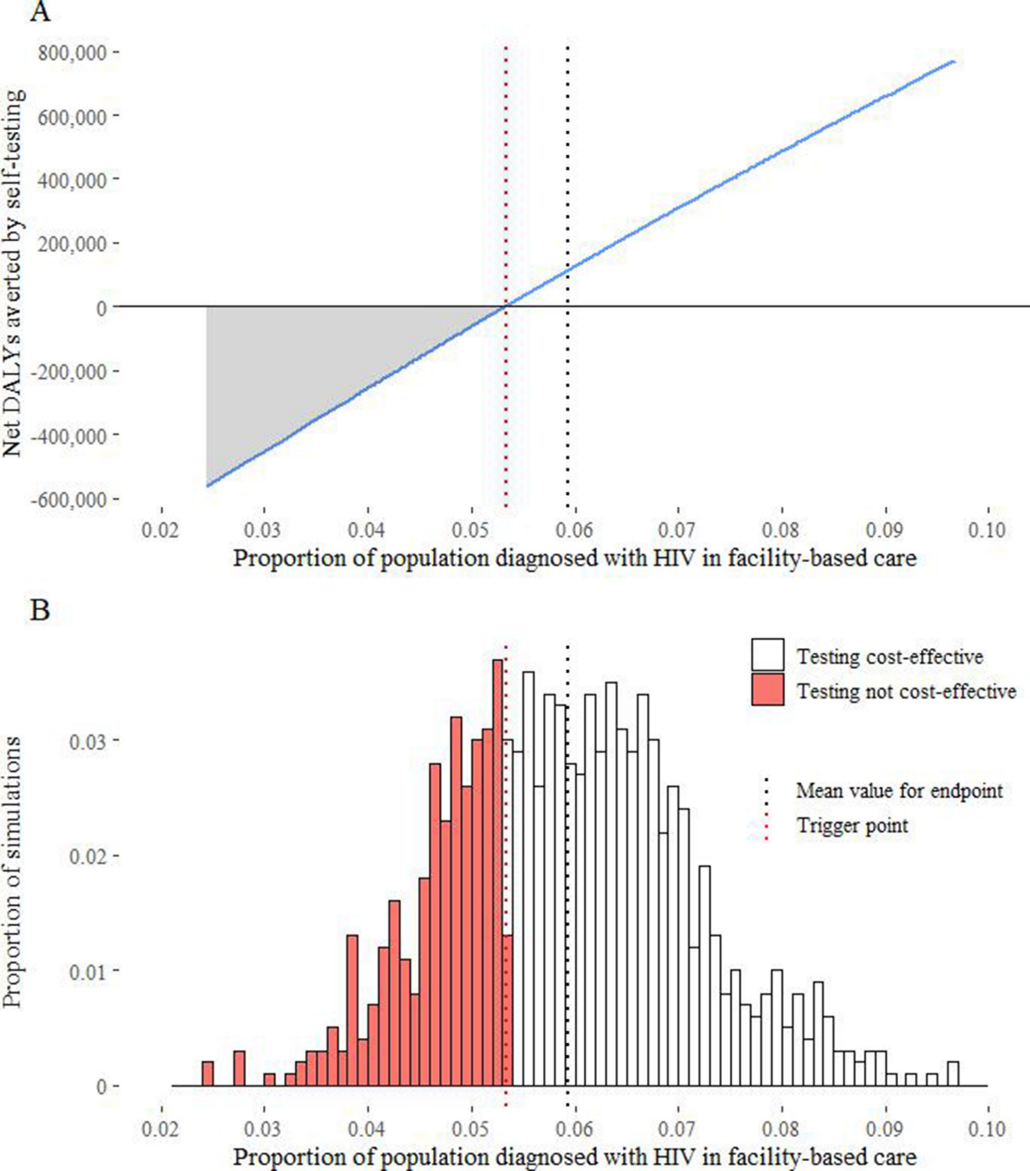
Calculating the value of a study of self-testing in women and men focused on outcomes. (A) shows the net health effects of the self- testing programme for different values of the outcome endpoint; (B) shows the prior on the outcome endpoint.

The implications of making decisions about the self-testing programme based on current evidence are shown in table 1. The self-testing programme is cost-effective as indicated by the ICER falling below the cost-effectiveness threshold of US$500/DALY averted and positive values for net DALYs averted. These results represent the expected net health effects of the programme. However, due to uncertainties in the evidence base, there is a possibility that self-testing is not cost-effective and in this case, implementing it will reduce net population health. Further research may, therefore, be of value to better understand the cost-effectiveness of the self-testing programme. Figure 3 shows how the principles outlined in figures 1–2 can be applied to quantify the implications of decision making based on further research on outcomes. The net DALYs averted by the self-testing programme increase as the proportion of people diagnosed with HIV increases (figure 3A). At the mean value of the outcome endpoint, the programme delivers net health gains (108 400 averted DALYs). If the proportion of people diagnosed with HIV is less than the trigger point of 0.05, then self-testing is no longer cost-effective. The probability that the outcome endpoint is below this trigger value is shown by the shaded area in figure 3B (probability of 0.33). If the outcome study is commissioned then this would avoid the programme being implemented in these circumstances. The avoided potential waste of healthcare resources can be translated into population health gain as indicated by the grey shaded area in figure 3A. Weighting these gains from research (figure 3A grey area) by the likelihood that the outcome endpoint takes these values (figure 3B red bars) shows that research could potentially avert an additional 41 700 DALYs compared with implementation without research.

**Table 1:** Population health consequences of implementation without and with additional research for the HIV self-testing case study

Implications of decision making without further research		
Expected DALYs averted by programme	1 884 832	
Expected additional long-term costs associated with programme	US$888 203 454	
Expected health opportunity costs of funding programme (DALYs incurred)	1 776 407	
Incremental cost-effectiveness ratio (US$/DALY)	US$471	
Expected net DALYs averted by implementation	108 425	
**Implications of decision making informed by further research**	**Outcomes study**	** Cost study**
Value of endpoint at which decision changes*	0.05†	US$9.98‡
Probability further research could change decision	0.33	0.33
Expected net DALYs averted via research	41 740	89 375
Potential maximum expenditure on study	US$20 870 062	US$44 687 606
Total expected net DALYs averted	150 165	197 800

*The units for this row are the proportion of the targeted population who are diagnosed with HIV in facility-based care for the outcomes study and the cost per person tested in US$ for the cost study.

†This indicates that expanded testing is no longer cost-effective if the proportion of the population who are diagnosed with HIV and linked to care is below 0.05.

‡This indicates that expanded testing is no longer cost-effective if the cost of testing exceeds US$9.98 per individual eligible for testing.

DALY, disability-adjusted life year.

The results of conducting this analysis for both the outcomes and cost endpoints are shown in table 1. The additional health benefits of research are 41 700 DALYs averted by the outcomes study and 89 400 DALYs averted by the cost study. The maximum amount a research funder should be willing to spend is US$20.9 million for the outcomes study and US$44.7 million for the cost study, this suggests that further research is potentially valuable in this setting.

So far we have shown how the population health benefits of research into self-testing can be quantified using existing models and model outputs and without use of advanced value of information methods. This does not answer a key question facing healthcare decision-makers, which is, when is the right time to implement a programme if we are uncertain about its net health effects?

Three different policy choices are available to healthcare decision-makers:

- Implementation without research: implement programme without further research if current evidence indicates it is cost-effective.
- Implementation alongside research: implement programme while conducting research, and consider scaling back programme if research shows programme does not improve net population health.
- Research then implementation: delay decision about implementation until research reports.

There are trade-offs to consider when choosing between these policies. If we wait until the research reports before implementing the programme, we forego the benefits of implementation in the meantime. On the other hand, if the programme is implemented while research is conducted there is a risk that the programme is found not to have been worthwhile once research findings emerge and is scaled back. This also risks the loss of resources where irrecoverable programme setup costs are high. In some cases, implementation alongside research may not be feasible.

We, therefore, quantified the net health consequences of each available choice open to policy-makers, assuming the outcomes study takes 3 years to report and the cost study 1 year to report. This analysis reflects that under the research then implementation policy there will be no access to self-testing in the research period, and reflects that under both policies involving a research component, the benefits of research do not emerge until the research reports. The methods for this part of the analysis are shown in the online supplementary material (see online supplementary material S3) and results are shown in table 2.

10.1136/bmjgh-2019-002152.supp2 Supplementary data

The outcomes study offers the potential to avert 12 400 additional DALYs if the programme is implemented alongside research. If programme implementation is delayed until research findings emerge then the net DALYs averted are 19 900 *lower* than if self-testing was implemented without further research. The benefits of having a programme up and running straight away (ie, implementation) exceed the benefits from making a more informed decision based on improved outcome data but delaying availability of the intervention by 3 years.

A cost study is expected to avert approximately 67 000 DALYs regardless of whether it is implemented alongside research or implementation is delayed until the cost study reports.

Our analysis underestimates the benefits of implementation alongside research for both the outcomes and the cost study. Without conducting additional analyses using the transmission model, we could not fully simulate the consequences of discontinuing the self-testing programme when research did not support continued implementation. This would have allowed the long-term benefits of the self-testing conducted in the 1 or 3 year research period to have been captured. Instead, we assumed that there were no further benefits of self-testing when the programme was discontinued. However, these potential missed benefits need to be weighed against set-up costs which will represent irrecoverable expenditures in the event that research suggests self-testing should be scaled back.

**Table 2:** Population health consequences of implementation and research policy choices

Implications of decision making without further research		
Expected net DALYs averted by implementation	108 425	
**Implications of decision making informed by further research**	**Outcomes study**	**Cost study**
**Implement testing programme alongside research**		
Expected net DALYs averted via research	12 376	66 380
Potential maximum expenditure on study	US$6 187 927	US$33 189 959
Total expected net DALYs averted	120 801	174 805
**Delay implementation until research reports**		
Expected net DALYs averted via research	−19 869	66 996
Potential maximum expenditure on study	−US$9 934 735	US$33 497 945
Total expected net DALYs averted	88 555	175 421

DALYs, disability-adjusted life years.

## Discussion

In this paper, we have shown how a graphical method can be used to estimate the value of research studies without advanced value of information methods. We provide a simple excel tool to allow readers to use the method. Where time and resources allow, information from existing studies, expert elicitation and outputs generated from existing cost-effectiveness models can be used to inform the calculations. Where the assembly of such information is not feasible, the method and tool can be used to test how different assumptions influence estimates of the value of research, identify the assumptions under which a proposed research study appears worthwhile, and allow decision-makers to consider their plausibility. These methods apply to a wide range of research studies aimed to inform programme design in the near-term (see online supplementary material S4) and can be used to quantify the value of collecting data on different endpoints and in different populations. The methods are relevant where evidence is expected to be considered relevant for decision making in multiple countries. The net health benefits of the research can be calculated for each country and considered collectively when assessing the value of the study.

When evaluating a specific research proposal, it may be important to consider, quantitatively or qualitatively, other factors that may modify the value of research. These include future changes that would modify the net DALYs averted by the intervention (eg, anticipated price reductions for health technologies), uncertainty around whether the research completes and is used to inform decisions, the degree to which uncertainty is reduced, and the potential for the study to generate additional secondary outcome data which may be used in a range of ways.^4^ In this example, we assumed the research study was small relative to the population that will benefit from the research and did not therefore account for the benefits of self-testing for those enrolled in the study. In some contexts, the population health benefits for this group are significant (eg, in the case of large studies) and could be included in the calculations.^40^

### Findings from the HIV self-testing case study

We applied the graphical method to a case study of HIV self-testing. This allowed us to show how existing evidence can be used to inform an assessment of the value of a future study, and how an assessment of the value of further research can be used to guide policy decisions relating to programme implementation and research.

This showed that a 1-year cost study is likely to be of high value, whereas a 3-year outcomes study offered more modest value. The outcomes study is only worth conducting if it is run in parallel with implementation. Delaying implementation until the outcomes study is complete results in worse outcomes than implementing self-testing without further research. Overall the results suggest that if a decision maker considered setup costs to be significant, they may wish to commission a cost study and delay implementation of self-testing until it reports. If setup costs are not considered significant, running a cost and outcomes study alongside implementation may be the preferred option.

The value of research is fundamentally an economic question, as research that aims to inform programme design can only deliver value if there is a chance that its results could change the assessment about whether a programme’s benefits outweigh the opportunity costs. The cost per DALY averted threshold used to determine the health opportunity costs imposed by programme costs is a key driver of this assessment. We used a value of US$500/DALY to reflect the opportunity cost of HIV service funding. This value is subject to uncertainty and our conclusions will differ if a different estimate of opportunity cost is used. This emphasises the need for both resource allocation and research prioritisation decisions to be based on a robust assessment of the opportunity cost of healthcare funds. Recent work has estimated the cost per DALY averted for general (ie, not HIV specific) healthcare spending in a range of countries.^13^ Using the estimate generated for Malawi of US$138/DALY^13^ within our analysis results in research no longer generating value. The health opportunity costs of dedicating funding to the self-testing programme become so high that even under optimistic scenarios about the outcomes and costs of testing, the programme will not produce positive net population health benefits. This may become relevant as funding of HIV services becomes more reliant on domestic rather than overseas funding.

The estimates presented reflect the impact of the self-testing studies for population health in Malawi. It is possible that the research could be used to inform resource allocation decisions in additional countries with similar local epidemiology and healthcare seeking behaviours. If this is the case, we will underestimate the value of the studies. Where a research study is expected to be used in a number of countries the approach described above can be extended to reflect the total global value of the research. The value of the study in each country can be estimated accounting for differences in the size of the population that stand to benefit from research, the costs and health benefits of the intervention and the cost-effectiveness threshold. This will generate estimate of the value of research in each country which can then be aggregated to estimate the global value of research.^4 41^ A worked example of this is provided in Woods *et al.*^42^

### Using estimates of the net DALYs averted by research to inform research prioritisation

Using estimates of the net DALYs averted by research to inform research prioritisation as suggested here is likely to require substantive changes to how evidence is used to support research funding decisions.

Currently, research funding decisions do not routinely use the type of evidence discussed in this paper. Institutional changes are required to facilitate use of the methods. This could include requiring funding bids to include these types of analyses, funders themselves conducting the analyses for submitted research bids, or decision-makers within LMICs conducting analyses to inform the specification of research calls. Research to explore how this might work in practise is ongoing in high-income settings^2 6 43^ and further work to assess this in LMICs is warranted.

Our case study focused on HIV where both evidence and detailed cost-effectiveness models are often well developed. In many contexts, models used to assess cost-effectiveness will be available and can be used and extended to make the value of information assessments described here. For decisions where available evidence is sparse, cost-effectiveness analyses unavailable, or collation of such evidence is not feasible, our work can be used to test the sensitivity of the value of research to different plausible assumptions. This may be sufficient to determine whether research should be funded. If decisions about research appear sensitive to different plausible assumptions then there may be value in low-level initial research funding to assemble existing evidence, conduct expert elicitation and develop basic cost-effectiveness analysis and make a more informed assessment of the value of research.

The robustness of any estimates of value of information will depend on the use of appropriate priors to represent uncertainties in the available evidence, the credibility of the underlying cost-effectiveness model, and use of an appropriate measure of health opportunity costs. Specification of each element is likely to require judgements regarding approach and input parameters. By using quantitative methods such as those set out here, the judgements are open to empirical challenge thus allowing for more accountable decision making. When cost-effectiveness analyses are used to support service investments this often involves an iterative process whereby relevant stakeholders review key judgements, and scenarios are presented exploring the implications of different judgements. We envisage a similar deliberative decision-making process could be usefully implemented when using value of information estimates to inform research prioritisation.

Ultimately, once an assessment of the potential population health benefits of a research study has been made, a research funder will have to assess whether the value offered by the research is sufficient to justify the opportunity costs imposed by funding the research. These opportunity costs depend on potential alternative uses for those research funds which may include other research and non-research investments. This raises the question of how the research funder should assess the opportunity costs of their research funds when prioritising between funding applications. One way of doing this is to ensure only those proposals with the lowest research cost per net DALY averted are funded, that is, a cost-effectiveness league table approach for research proposals. The net DALYs averted by research estimated using the methods presented here could be used alongside the research costs to generate this information. In the absence of this evidence, a useful but imperfect starting point is to assume that the opportunity cost of research funds and service funds is similar. We have used this assumption within this work to estimate the maximum a research funder should be willing to spend on a study. Where research costs are known this assumption can be used to translate research costs to health opportunity costs which can be directly compared with the net health benefits of research. Those proposals offering the largest difference between net health benefits of research and health opportunity costs of research funding may be considered particularly attractive to research funders.

## Conclusion

Our work provides a method for estimating the health benefits of research in a practical and timely fashion. This can be used to prioritise funding of those research and evidence generation activities that offer real potential to improve population health.
