# Supplementary material for: Practical metrics for establishing the health benefits of research to support research prioritisation
Source: BMJ Glob Health. 2020 Aug 31;5(8):e002152. doi: 10.1136/bmjgh-2019-002152 (PMC7462234; doi:10.1136/bmjgh-2019-002152)
Supplement: Supplementary data [file bmjgh-2019-002152supp001.pdf]

Supplementary material

S1 Priors for study endpoints

The prior for the proportion of the targeted population linked to and diagnosed within facility-based care was based on a systematic review and meta-analysis by Sharma et al.[1] This looked at a wide range of HIV testing approaches in sub-Saharan Africa. Analyses restricted to self-testing were used where available. The systematic review provided data for three related endpoints: uptake which is calculated as the proportion tested of those offered testing, HIV positivity which is calculated as the number of individuals testing HIV positive of those tested, and the proportion linked to facility-based care of those who tested positive (we assume that all of these individuals will then be diagnosed with HIV). Estimates of the mean for each endpoint, and the associated confidence intervals, are presented in Table S1. A beta distribution was used to represent the prior for each endpoint.[2] The standard errors for the distributions were doubled to reflect the additional uncertainty inherent in generalising data from the literature to a future specific study setting. The overall prior for the proportion of the targeted population ultimately linked to and diagnosed within facility based care was then estimated by randomly sampling from each beta distribution and multiplying the three sampled proportions.

Table S1: Data used to inform prior for the proportion of the targeted population linked to facility-based care

**Parameter**	**Proportion:**<br>**Mean (95% confidence interval)**	**Notes on source**
Uptake	0.69 (0.59-0.78)	Random effects meta-analysis of home self-testing studies from Sharma et al. Reflects two studies conducted in Malawi including three study arms.
HIV positivity	0.09 (0.08-0.10)	Restricted to facilitated linkage study arm as most studies of self-testing are now focused on introducing some form of enhanced linkage, and this is expected to impact on reporting of positive HIV test results.
Proportion linked to facility-based care	0.95 (0.87-0.98)	Endpoint not reported for self-testing studies. Data instead reflect home-based and campaign community testing with counselling and facilitated linkage.

In 2014/15, when a number of self-testing studies were being designed, there was very limited data relating to costs for self-testing programmes. The review by Sharma et al. did not identify any costing studies for self-testing but did report costing data for home-based community HIV testing

and counselling. These estimates ranged from $7.2 to $14.65 per person tested (data in 2012 USD and collected in Kenya, Malawi, Swaziland and Uganda). We assumed that given the potential savings in time from self-testing that $15 represented an appropriate upper bound on testing, and $3 per person represented an appropriate lower bound. We therefore used a gamma distribution with mean $9 and confidence interval $3-15 for the cost of the self-testing programme per individual targeted for testing.

S2 Estimating the relationship between net DALYs averted and key endpoints

The relationship between net DALYs averted and the cost and outcome endpoints were informed by existing scenario analysis of the HIV synthesis model. We included in our analysis those scenarios modelled by the HIV synthesis model which are representative of the situation in Malawi. This was achieved by selecting scenarios where the calibration scores indicated sufficient agreement between the model predictions and key data on the Malawian context.

The existing scenario analyses were used as the basis for this work. An alternative approach would have been to run additional scenarios which better fitted our purpose (e.g. one-way sensitivity analyses examining the impacts of the cost and outcomes endpoints). Although this would have simplified our work this would have created additional burden for the HIV synthesis modelling team due to the large amount of analyst and computational time taken to run the model.

Regressions were developed using the scenario analysis from the HIV synthesis model as data. These regressions used the scenario analysis outputs to predict DALYs averted by expanded testing and total costs incurred by expanded testing (compared to the standard of care core testing) as a function of the endpoints of interest. The regression analysis for DALYs averted included the proportion of the targeted population who are diagnosed with HIV in facility-based care in year 0-3 (the outcome endpoint) as an independent variable. The regression analysis for total costs incurred included both the outcome endpoint, and programme cost per targeted individual (the cost endpoint) as independent variables. Regression models were fitted that allowed DALYs and total costs to be a non-linear function of the endpoints and that allowed the endpoints to interact. Constraints were imposed on the regressions so that DALYs averted must increase with the outcome endpoint, and costs incurred must increase with this endpoint and the cost endpoint. These constraints are in recognition of some residual stochastic variation in the model simulation outputs and avoid the flexible model used over-fitting to individual scenario results and producing implausible results. The analysis used the Shape Constrained Additive Models package in R.[3]

There was wide variation across testing scenarios in the number of individuals targeted for testing i.e. the scale of the testing programme. This reflects the objective of the original analysis which aimed to explore a wide range of testing programmes with different characteristics. Across scenarios, variation in programme scale was correlated with the outcome endpoint. This was intended to reflect that smaller programmes are often expected to have higher yields of HIV positive individuals (e.g. partner testing). Uncertainty in the size of the target population is not however something we expected to address via the cost or outcome studies we are evaluating. We therefore rescaled the total population DALYs and total costs to per capita values prior to conducting the regression analysis.

The regression analysis allowed direct prediction of the DALYs averted and total costs incurred per capita for different values of the endpoints of interest. Predictions were made for 1,000 values simulated from the prior for the outcome endpoint and 1,000 values from the prior for the cost endpoint. Predictions of DALYs averted and total costs incurred were used alongside the estimate of the cost-effectiveness threshold to estimate net DALYs averted by self-testing compared to the core testing programme for different values of the endpoints.

To estimate the value of the outcomes study we required an estimate of net DALYs averted for each value of the outcome endpoint. This was calculated by taking each value of the prior for this endpoint, and then averaging the net DALYs averted across the values for the prior for the cost endpoint. This averaging reflects the fact that if we run an outcomes study the cost endpoint will remain uncertain. The resulting information on the relationship between the outcome endpoint and net DALYs averted was then used alongside the prior for the outcome endpoint to estimate the value of the outcome study as shown in the main manuscript (see Figure 1 and surrounding text). An equivalent process was followed to estimate the value of the cost study. Expected outcomes in the absence of further research were calculated by average DALYs averted and additional costs across values of the endpoints. This ensures that the estimates underpinning the analysis of decision making without further research are the same as the estimates underpinning the analysis of decision making informed by further research.

The per capita estimates of net DALYs averted by research were then rescaled to reflect the average population eligible for the testing programme from the HIV synthesis model. This was calculated by fitting a polynomial describing the relationship between the population eligible for testing and the outcome endpoint. The predicted population size was then generated for each value of the outcome endpoint sampled from the prior. These values were then averaged to calculate the average eligible population.

S3 Impact on net DALYs averted by research of delaying implementation until research reports or implementation alongside research

Implementation alongside research

When a programme is expected to be cost-effective based on currently available evidence, a decision maker may consider implementing the programme whilst the research is conducted. This allows the expected benefits of implementation to accrue whilst the research is being conducted. From the point that research is reported, if research supports continued investment in the intervention the benefits and costs continue to accrue for the remainder of the model time horizon. If the research shows the programme is not cost-effective then the programme can be cancelled to avoid further negative impacts on net population health. For example, if research takes 3 years to report the expected benefits of implementation accrue over years 0-3 and the benefits of research accrue over years 3-50.

These principles are reflected in the analysis by running two sets of regressions to predict DALYs averted and costs incurred:

(1) analyses for full model time horizon of 50 years; and

(2) analyses removing those benefits or costs accrued during the initial research period of 1 or 3 years, depending on the study.

The difference between the expected net DALYs averted using regression set (1) and the expected net DALYs averted using regression set (2) can be used to calculate the expected net DALYs averted by implementation during the 1- or 3-year research period. The results from analysis (2) can be used to calculate the value of implementation informed by research and will reflect the value from the point that research reports up to the model time horizon. The sum of these two components represents the total net health effects of the implementation alongside research policy.

The scenarios from the HIV synthesis model reflect two possible situations, implementation of additional testing or continue with standard of care core testing. Under the implementation alongside research scenario we have a third situation where testing may be implemented for 1 or 3 years during research but then cancelled if the research does not support continued implementation. We did not have access to an analysis modelling the implications of this and therefore assumed that there were no further consequences of self-testing beyond years 1 or 3 in those scenarios where the self-testing programme was discontinued. This is a simplification as there may be costs associated with winding down the programme and the costs and health benefits for those diagnosed during the research period will continue to accrue beyond this period. For further

of discussion of this issue and potential additional analyses that could be conducted see Woods et al.[4]

Research then implementation

Health care decision makers may choose to wait until they know the results of research before implementing the programme. This delay will affect the net DALYs we can expect to avert by using the research to make more informed decisions. When implementation is delayed until research reports the intervention delivers no benefit (outside of the research population) until research reports. The net DALYs averted by research therefore accrue over a shorter time horizon than the full 50 year model time horizon. This is reflected in the analysis by reducing the time horizon of the model by the duration of the research (1 year for costing research, 3 years for outcomes). The net DALYs averted and total costs incurred under each scenario were therefore computed using a 47 year and a 49 year time horizon as well as a 50 year time horizon. The value of research could then be computed using these revised figures within the regression and subsequent analyses described in section S2. The resulting value of research was then subjected to additional discounting to reflect the fact that beneficiaries of the intervention would now have to wait an additional 1-3 years to receive it.

Approach for settings where disease dynamics are not considered relevant

The approach described above applies to a population-level model that reflects use of a programme over time. These types of model are often used where disease dynamics are considered an important determinant of the impacts of interventions.

An alternative, simpler, approach can be used where it is considered appropriate to characterise the impact of interventions at the individual or cohort level without consideration of disease dynamics. This is typically the case for non-communicable diseases, or interventions that are used in the context of infectious diseases but are not expected to modify disease dynamics. In such context's individual-level estimates of net health effects can be rescaled to reflect use of an intervention in different numbers of individuals in a population or over time.

To illustrate this, we use a numeric example where 1,000 people per year are eligible for the intervention and the intervention will be used for the next 20 years, we also assume a discount rate of 3%. From the tool we can calculate the per capita net DALYs averted when decisions are made based on current evidence and when further research is used to support decision making. We

assume this has been done and shows that under current evidence implementation would result in an expected gain of 1 net DALY averted per capita and that decision making informed by further research would generate an expected 1.5 net DALYs per capita. We also assume research takes 5 years to report. To generate the outcomes of each policy (implementation without research, implementation alongside research and research then implementation) we proceed as shown in Table S2.

Table S2: Estimating the overall population health implications of implementation without research, implementation alongside research and research then implementation

	Undiscounted net DALYs averted			Discounted net DALYs averted		
Year	Implementation without research	Implementation alongside research	Research then implementation	Implementation without research	Implementation alongside research	Research then implementation
1	1,000	1,000	-	1,000	1,000	-
2	1,000	1,000	-	971	971	-
3	1,000	1,000	-	943	943	-
4	1,000	1,000	-	915	915	-
5	1,000	1,000	-	888	888	-
6	1,000	1,500	1,500	863	1,294	1,294
7	1,000	1,500	1,500	837	1,256	1,256
8	1,000	1,500	1,500	813	1,220	1,220
9	1,000	1,500	1,500	789	1,184	1,184
10	1,000	1,500	1,500	766	1,150	1,150
11	1,000	1,500	1,500	744	1,116	1,116
12	1,000	1,500	1,500	722	1,084	1,084
13	1,000	1,500	1,500	701	1,052	1,052
14	1,000	1,500	1,500	681	1,021	1,021
15	1,000	1,500	1,500	661	992	992
16	1,000	1,500	1,500	642	963	963
17	1,000	1,500	1,500	623	935	935
18	1,000	1,500	1,500	605	908	908
19	1,000	1,500	1,500	587	881	881
20	1,000	1,500	1,500	570	855	855
**Total**	**20,000**	**27,500**	**22,500**	**15,324**	**20,627**	**15,910**

S4 Research studies informing programme design and resource allocation

Table S3 shows the range of studies to which the methods presented can be applied, and provides information on some of the funders who support these studies and who may therefore be interested in quantitative assessments of their health effects. The table also indicates the potential generalizability of different types of studies. This will have implications for the extent to which there is a need to assess the health benefits of the studies in different countries and aggregate total value across countries.

Table S3: Research and evidence generation activities to support HIV treatment and prevention programmes that could be evaluated using these methods

Types of studies	Example outputs used to inform decision making	Expected generalisability of research across settings	Example funders
Phase III-IV clinical trials of medical interventions (drugs, diagnostics, vaccines)	• Effectiveness, e.g., in terms of individual health outcomes or acquisition of HIV;<br>• Measures of feasibility and cost of service implementation	High or medium	European & Developing Countries Clinical Trials Partnership (EDCTP)<br>UK Medical Research Council (MRC)<br>Wellcome Trust<br>US National Institutes of Health (NIH)
Implementation studies	• Impact of alternative models of delivery of care on programme engagement and costs in different populations and geographies.	Low or medium	US NIH Fogarty International Center<br>Population Council<br>President's Emergency Plan for AIDS Relief (PEPFAR)<br>U.S. Agency for International Development (USAID)<br>Bill and Melinda Gates Foundation
Epidemiological studies including surveillance studies and longitudinal follow-up	• Prevalence and incidence of HIV and their variation across time, place and subpopulations;<br>• Behavioural surveillance measures (e.g. number and nature of sexual partners, use of condoms);<br>• Programmatic data on number of individuals receiving specific treatments or prophylaxis;<br>• Response to antiretroviral therapy (viral load, CD4 counts, resistance), rates of clinical events, including mortality, rate of loss to follow-up and re-engagement in care, and how these vary across geographies and subpopulations (defined by socioeconomic status, disease features, urban/rural etc.).	Low or medium	Nationally funded programme monitoring and surveillance data.<br>USAID (funds Demographic and Health Surveys, DHS, alongside other international and national funders)<br>US Centers for Disease Control and Prevention (CDC) (funds Population-based HIV Impact Assessments (PHIA) surveys)<br>UK MRC<br>UK Department for International Development<br>Wellcome Trust<br>Bill and Melinda Gates Foundation
Resource use and cost studies	• Programme costs and how these vary across geographies, by subpopulation, by programme scale, and by service delivery modalities. May be integrated into trials or implementation studies.<br>• Costs of long-term disease management.	Low	Bill and Melinda Gates Foundation
User perspectives and preferences including morbidity surveys	• Disability weights (for computation of disability-adjusted life years, DALYs or quality-adjusted life years, QALYs)<br>• Out of pocked and indirect costs of health care use	Low	Bill and Melinda Gates Foundation

Supplementary material references

1. Sharma M, Ying R, Tarr G, et al. A systematic review and meta-analysis of community and facility-based approaches to address gaps in HIV testing and linkage in sub-Saharan Africa. Nature. 2015;528(7580):S77.
2. Briggs A, Sculpher M, Claxton K. Decision modelling for health economic evaluation: OUP Oxford; 2006.
3. Pya N. scam: Shape Constrained Additive Models. 2018.
4. Woods BS, Rothery C, Revill P, et al. Setting research priorities in Global Health: Appraising the value of evidence generation activities to support decision-making in health care. Centre for Health Economics, University of York, York.: 2018.
